# Stress associated with hospitalization in patients with COPD: the role of social support and health related quality of life

**DOI:** 10.1186/2049-6958-7-51

**Published:** 2012-12-10

**Authors:** Magdalena Medinas-Amorós, Juan José Montaño-Moreno, Maria José Centeno-Flores, Victoria Ferrer-Pérez, Feliu Renom-Sotorra, Belen Martín-López, Catalina Alorda-Quetglas

**Affiliations:** 1 Psychology Department, Joan March Hospital, Ib-Salut, Cra. Palma-Soller Km.12.5. Bunyola, Mallorca, Balearic Islands, Spain, CP: 07193; 2 Psychology Department, University of the Balearic Islands, Mallorca, Balearic Islands, Spain; 3 Department of Respiratory Nursing, Joan March Hospital, Ib-Salut, Mallorca, Balearic Islands, Spain; 4 Department of Respiratory Medicine, Joan March Hospital, Ib-Salut, Mallorca, Balearic Islands, Spain

**Keywords:** Hospitalization, Quality of Life, Social Support, Stress

## Abstract

**Background:**

The objective of this study was to determine stress levels during hospitalization in patients with Chronic Obstructive Pulmonary Disease (COPD). We wanted to relate stress to previous level of quality of life and patients’ Social Support.

**Methods:**

80 patients (70.43; SD = 8.13 years old) with COPD were assessed by means of: Hospital Stress Rating Scale, Nottingham Health Profile, St. George’s Respiratory Questionnaire and Social Support Scale.

**Results:**

COPD patients’ stress levels are lower than expected independently from the severity or number of previous hospitalizations. Linear regression analysis shows the predictive value of Quality of Life and Social Support on stress level during hospitalization (p < 0.0001).

**Conclusion:**

HRQOL and social support can be associated with stress during hospitalization.

## Background

The physical and social configuration of hospitals implies the possibility of introducing inpatients to adverse psychological effects. These effects have been described and analyzed by different authors over the past few years [1-4]. Volicer et al [5]. identified hospital experiences related to routine attention that are perceived as stressful during hospitalization, like: difficulties to obtain data concerning their own therapeutic processes, ignorance of the consequences of the disease, dangers that test involves, reduction of intimate space in the room, use of medical language, restriction of visits of own family’s members and friends, etc. Based on these observations the authors drew up the 40 items questionnaire called Hospital Stress Rating Scale. This scale was validated and translated in Spanish by Kendall [6]. Between 1977 and 1978 other studies on stress during hospitalization incorporated the importance of cardiovascular and socio-demographic factors during hospitalization [7-9], coping strategies and psychological impact [10,11] and severity of the disease, and presence of pain as important variables, affirming that some of them can predict stress level during hospitalization [12] in acute patients. During the 80s, authors focused their interest on factors related to the patients’ difficulties of adjustment to hospital [13-15]. Researches carried out over the two last decades have extended the study of the iatrogenic effects of hospitalization to different pathologies [16,17] or stress perception differences between patients and health care staff [11,18,19].

Chronic Obstructive Pulmonary Disease (COPD) is one of the most prevalent respiratory diseases with a frequent need for hospitalization [20]; these patients suffer acute exacerbations during the year and hospitalization is an important part of patient care. The illness severity and a progressive loss of quality of life and physical mobility deteriorate patients’social and family support [21]. Nevertheless, although poor relations between pulmonary function and quality of life and social support have been reported in the literature [22], recent studies have shown that improving physical activity (specific rehabilitation programs) can reduce symptoms and associated psychological dysfunctions and increase well-being and quality of life in these patients [23,24].

Currently, a bibliographic review of databases does not show scientific studies concerning stress or the psychological effects of hospitalization in patients with COPD or in patients with chronic diseases who need frequent hospitalization. Andenaes et al [25] observed high levels of stress in hospitalized COPD patients, concluding that 58.7% of them showed different levels of stress in comparison to non-hospitalized COPD patients. Then, in their follow up study, Andenaes et al [26] observed a decrease in stress levels nine months after hospitalization; so the authors deduced that this had clearly been caused by hospitalization.

In a previous study, we can observe that patients with COPD suffer low stress levels during hospitalization [27] but it is not clear the role of Health Related Quality of Life (HRQOL) and Social Support. For this reason, the aim of this study is to determine stress levels during hospitalization in patients with a chronic disease such as COPD. After that, we want to relate these parameters to the previous level of quality of life and patients’ social support. We have hypothesized that these last two parameters can contribute to diminishing stress levels in COPD patients.

## Methods

### Study design and patients

A cross-sectional sample of patients with COPD was selected and examined comprehensively with lung-function tests and a battery of specific scales described in the measures section. All patients were inpatients and were assessed after 10 days of hospitalization. This study was approved by the Ethical Committee at the Department of Research of GESMA, Balearic Islands, Spain. All patients signed the Informed Consent before the study. Finally, 80 consecutive patients (65 men and 15 women) with a diagnosis of COPD who agreed to participate and who met the Inclusion Criteria were included in our study. Inclusion criteria were as follows: COPD with a ratio of FEV_1_/forced vital capacity (FVC) < 0.7, FEV_1_ < 80% of predicted, age 45–90 years. Exclusion Criteria were : another disabling or severe disease that could prevent carrying out all study tests, and/or dementia. Patients with an indicative history of asthma were excluded. No patient had an acute COPD exacerbation at the time of the research.

### Clinical data and lung-function tests

All participants were administered a structured clinical interview to obtain the following clinical, social and demographic parameters: age, gender, occupation, education level, degree of severity of COPD based on spirometry, degree of dyspnoea [28] (MRC, Medical Research Scale), number of hospitalizations during the previous year and comorbidities by Charlson Index [29]. Assessment of lung function (FEV1, FVC, absolute and reference values) was based on the 2006 reference spirometry values from Global Initiative for Chronic Obstructive Lung Disease (GOLD Classification).

### Questionnaires and scales

**Hospital Stress Rating Scale**[5]**(HSRS):** made up of a list of 49 commonly stressful hospital events assessed by patients (9 points: 1 “not at all stressful”, 9 “extremely stressful”) as to whether he or she had experienced it. We applied the author’s score correction (items summation); range of scores was 49 to 441 points.

**The Nottingham Health Profile**[30]**(NHP):** The NHP was developed for General Quality of Life measurement. It consists of two parts. Part I contains 38 yes/no items in six dimensions: pain, physical mobility, emotional reactions, energy, social isolation, and sleep. Part II contains seven general yes/no questions on daily living problems. The two parts may be used independently, and part II has not been analyzed in this study. The NHP questionnaire has an adapted, validated Spanish version [31], which was used in our study. A higher NHP score shows a worse quality of life.

**The St. George’s Respiratory Questionnaire (SGRQ)**[32]^**:**^ is a standardized self-administered airways disease-specific questionnaire. This questionnaire has an adapted and validated Spanish version used in our study [33]. It contains 50 items divided into three subscales: symptoms (8 items), including several respiratory symptoms, their frequency and severity; activity (16 items), on activities that cause or are limited by breathlessness; and impact (26 items) which covers a wide range of aspects related with social functioning and psychological disturbances resulting from airways disease. The higher is the SGRQ score the lower the general quality of life.

**The Medical Research Council Scale (MRC)**[28]: comprises 5 self-administered statements that describe respiratory disability (Dyspnoea) from none (Grade 1) to almost complete incapacity (Grade 4).

**MOS Social Support Survey**[34]**(MOS).** A total of 19 functional support items were developed to assess perceptions of the availability of different functional aspects of support. Subscales of MOS are: Emotional support (4 items), Informational support (4 items), Tangible support (4 items), Affectionate support (3 items) and Positive social interaction (4 items). Higher scores correspond to more social support. The MOS Social Support Survey, Spanish version, was identical to the original version.

### Procedure

Sample selection and later information collection were carried out in the Pneumology Unit of the Joan March Hospital in Majorca, Spain. The final sample was made up of patients diagnosed with COPD, selected consecutively according to when they were admitted to the afore-mentioned hospital. Patients were assessed during the next 10 days, once their clinical state had been stabilized. All patients were assessed by means of two interviews. During the first interview, socio-demographic, clinical data and Informed Consent were registered. In a second interview, the above scales and questionnaires were administered. All assessment was carried out in the patient’s room without a companion to guarantee confidentiality and correct administration of the questionnaires.

### Statistical analysis

Data were analyzed by means of statistical program SPSS 17. For the socio-demographic and clinical data and MRC a descriptive study was conducted. For total stress scores, quality of life and social support variables, the variable’s distribution was analyzed before multiple comparison tests in order to verify our hypothesis. For Multiple Regression analysis (stepwise method), only normal distribution variables were selected. The significance level used was p < 0.05 (Confidence Interval: 95 %).

## Results

A sample of 80 patients with a mean age of 70.43 years (SD = 8.13), range 49–87 years, were included. Socio-demographic characteristics reveal that patients are mainly married/partner men (52.1%), retired (47.9%), with a medium level of studies (65.15%), who live with their families (69.68%). Clinical data are showed in Table 1.

**Table 1:** Clinical characteristics of the sample (n = 80)

**Variable**	**Mean**	**SD**
**FVC**	63.58	19.65
**FEV1 % ref.**	36.89	16.20
**Basal SaO2**	92.40	2.87
**MRC**	3.01	0.76
**Number of previous hospitalizations/year.**	3.29	1.31

The HSRS results show a lower value than the mean score of the HSRS questionnaire (range of scores: 49 to 441 points) [5]; HSRS mean score: 220.5; COPD patients mean score: 166, 4; difference: 54.1 points. COPD patients consider the items related to change of habits, loss of control, loss of autonomy and privacy, as the most stressful items during hospitalization.

**Figure 1:**
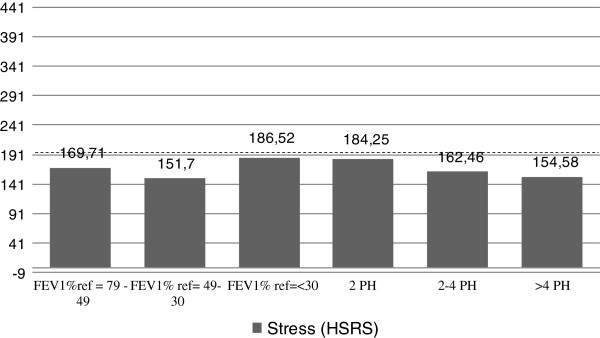
**Stress level results (HSRS) based on severity and number of previous hospitalizations/year (PH).** FEV1% pred.: Forced expiratory volume in 1 second (FEV1) % predicted; PH: Number of previous hospitalizations/year.

The comparative analysis of clinical severity variables, based on Gold classification, number of previous hospitalizations and MRC scale don’t show significant differences in stress level (Figure 1).

A descriptive study of the HRQOL and MOS questionnaires shows that these were bad in all COPD patients (Table 2). Table 3 shows the significant (p < 0.05) results based on NHP, MOS and SGRQ comparisons. The division into “High” and “Low” scores has been based on the statistical study of the obtained scores, placing the point of court in the percentile 50. The percentile 50 has been calculated for every questionnaire.

**Table 2:** Results of HSRS, HRQOL (SGRQ and NHP) and MOS questionnaires for COPD patients (n = 80)

**Instrument**	**Subscale**	**Min.**	**Max.**	**Mean**	**S.Err.**
**HSRS**	Total score	49	441	166.4	36.89
**SGRQ**	Symptoms	19.68	85.70	57.329	17.180
	Activity	22.30	100.00	66.973	21.514
	Impact	16.96	89.20	46.542	18.844
	**Total score**	**25.53**	**86.89**	**55.685**	**15.689**
**MOS**	Emotional support	14.00	40.00	29.411	8.658
	Tangible support	7.00	24.00	16.843	4.328
	Positive social interaction	4.00	20.00	13.764	4.572
	Affectionate	3.00	15.00	10.921	4.117
	**Total Score**	**35.00**	**95.00**	**70.941**	**19.153**
**NHP**	Energy	0.00	100.00	63.010	33.746
	Pain	0.00	100.00	23.589	34.361
	Physical mobility	10.80	88.80	59.057	22.911
	Emotional reactions	0.00	100.00	30.644	28.922
	Sleep	0.00	100.00	44.464	33.588
	Social isolation	0.00	87.00	22.285	23.869
	**Total Score**	**6.00**	**81.68**	**40.161**	**18.402**

**Table 3:** Stress level (HSRS) based on NHP SGRQ and MOS subscales (only p < 0.05 results)

**Variable**	**Mean HSRS**	**Stand. Error**	**Levene test (p)**	**Student test (p)**
**Total Score NHP**				
High HRQOL	161.064	8.996	F = 0.087 (p = 0.769)	t = −2.003 p = 0.050
Low HRQOL	179.400	12.107		
**Physical Movility (PM)**				
Without PM	145.600	6.888	F = 8.692 (p = 0.005)	t = −3.381 p = 0.002
With PM	190.038	11.192		
**Pain**				
No pain	149.931	6.697	F = 7.597 (p = 0.010)	(t = 2.694) p = 0.009
With pain	192.409	12.831		
**SGRQ Total Score**				
High HRQOL	150.000	8.273	F = 2.672 (P = 0.202)	t: -2.597 p = 0.012
Low HRQOL	185.807	10.930		
**Impact**				
Low impact	151.160	8.254	F = 2.915 (P = 0.094)	t = −2.412 p = 0.020
High impact	184.692	11.083		
**MOS Total Score**				
Without Social Support	192.200	16.482	F = 4.078 (P = 0.049)	t: 2.204 p = 0.032
With Social Support	158.277	7.228		
**Positive Social Interaction (PSI)**				
No PSI	200.300	18.294	F = 1.201 (P = 0.279)	t: 2.262 p = 0.028
With PSI	160.439	7.496		

**Linear Regression**: normal distribution variables were selected: clinical variables (FEV1% ref., and previous hospitalizations), NHP variables (Pain, Physical Mobility and Total Score), SGRQ variables (Impact and Total Score), MOS (total score and Positive social interaction) and MRC scale. The final regression model (Table 4) shows that this model predicts 55, 2% (p < .001) of the variability.

**Table 4:** Results of Linear Regression analysis (stepwise method) with Stress (HSRS) as dependent variable and NHP, SGRQ, MRC and MOS as independent variables

	**UnSd Coeff.**		**Sd Coeff.**				
**MODEL**	**B**	**SE**	**Beta**	**t**	**(p)**	**R**	**R**^**2**^
(Constant)	150,074	7,905	-	18,985	0,000*	0,490	0,240
· HNP Pain	0.741	0.188	0.490	3.931	0.000*		
(Constant)	207,651	25,299	-	8,208	0,000*	0,566	0,320
· NHP Pain	0.835	0.184	0.552	4.529	0.000*		
· MRC	−19.832	8.317	−0.290	−2.385	0.021*		
(Constant)	198,739	24,341	-	8,165	0,000*	0,631	0,398
· NHP Pain	0,639	0,192	0,423	3,322	0,002*		
· MRC	−30,416	9,010	−0,446	−3,376	0,001*		
· Impact (SGRQ)	0,981	0,399	0,356	2,456	0,018*		
(Constant)	195,336	22,422	-	8,712	0,000*	0,708	0,501
· NHP(Pain)	0,500	0,183	0,331	2,738	0,009*		
· MRC	−30,114	8,290	−0,441	−3,633	0,001*		
· Impact (SGRQ)	1,485	0,402	0,538	3,692	0,001*		
· Social Isolation(NHP)	−0,787	0,255	−0,361	−3,086	0,003*		
(Constant)	244,436	30,583	-	7,993	0,000*	0,743	0,552
· NHP(Pain)	0,335	0,190	0,222	1,768	0,084		
· MRC	−31,424	7,966	−0,460	−3,945	0,000*		
· Impact (SGRQ)	1,523	0,386	0,552	3,948	0,000*		
· Social Isolation(NHP)	−0,915	0,251	−0,420	−3,648	0,001*		
· Positive Social Interaction (MOS)	−2,908	1,289	−0,256	−2,256	0,029*		

*p < 0.05.

## Discussion

Stress is a mental and physical strain due to threats, danger, life changes and everyday challenges. Our study shows that COPD patients hospitalized in this chronic hospital perceived hospitalization as a little stressing event, contrary to prior literature findings already exposed. However, these hospitalized chronic patients were exposed to potentially stressing factors and suffered from the same effects of routine and protocols, consolidated throughout their history in these institutions [5,6] which can affect disease evolution and prognosis. Our analysis confirms that, in COPD patients, the most powerful stress factors were related to environmental factors (for example remaining in the same room and sleeping in a bed different from the habitual one); however, in previous studies, these items occupied the last positions in hospitalized patients in general hospitals [5,18,19].

One factor that seems important to explain these results is the chronic condition and previous experiences of our patients. Studies by Becker [35] and Gamarra [36] clearly differentiate acute patients from chronic patients, establishing that in the latter ones the increase in care demands and the necessity to be protected become a high-priority for them. Specially elderly hospitalized COPD patients need continuous health care and consider the hospital for chronic patients as a place with high protection against the consequences of their disease. In fact, our results show that patients with a lesser degree of dyspnoea and more self-care independence, reported the greatest stress levels and patients with severe dyspnoea were less affected by hospital routine [37].

### HRQOL and social support

A descriptive study of specific HRQOL for COPD shows that, on the whole, this was impaired in COPD patients. However, patients with a worse HRQOL experienced more stress during hospitalization than those who presented a better HRQOL.

## Conclusions

We believe that our study adds evidence to the association between stress during hospitalization and factors like pain, psychological impact of the disease, dyspnoea, social isolation and social positive interaction in COPD patients. These factors are shown to be good predictors of the stress during hospitalization. Our findings are consistent with the studies by Fernandez et al [38], McCathie et al [39] and Martin et al [40] while providing new insights into the psychological complications that may occur in patients with low levels of social support during hospitalization.

However, our study presents several limitations. Firstly, the sample size was relatively small, due to the difficulties to include elderly, clinically stable patients and difficulties for exhaustive psychological evaluation in a restricted context. Secondly, there are few research papers and too old scientific evidence that examine stress factors both in general and in patients with COPD.

## Abbreviations

COPD: Chronic Obstructive Pulmonary Disease; HRQOL: Health Related Quality of Life; HSRS: Hospital Stress Rating Scale; NHP: Nottingham Health Profile; SGRQ: St. George’s Respiratory Questionnaire; WT: Walking Test.

## Competing interests

The authors declare that they have no competing interests.
